# Genetically Informed Single-Cell Analysis Reveals *PLXND1* as a Cell-Type-Specific Molecular Switch in MASLD

**DOI:** 10.3390/metabo16060378

**Published:** 2026-05-30

**Authors:** Xianyi Ma, Junbo Song, Xin Hong, Zhibin Lin

**Affiliations:** Department of Hepatobiliary Surgery, Xijing Hospital, Fourth Military Medical University, Xi’an 710032, China; xianyima2025@163.com (X.M.); songjunbo2009@163.com (J.S.); qpzmhx@163.com (X.H.)

**Keywords:** *PLXND1*, MASLD, Mendelian randomization, single-cell transcriptomics, genetic causality, lipid-associated macrophages, precision medicine

## Abstract

**Background/Objectives:** Metabolic dysfunction-associated steatotic liver disease (MASLD) is a systemic disorder driven by genetic predisposition, epigenetic programming, metabolic rewiring, and immune dysregulation. Although population genetics and single-cell transcriptomics have advanced our understanding, the multi-omic causal architecture of MASLD at cellular resolution remains poorly defined. This study aimed to establish an integrative framework linking genetic causality to cell-type-specific tissue dysfunction. **Methods:** Multi-layered Mendelian randomization (MR) and summary-data-based MR (SMR) across large-scale eQTL and pQTL datasets were applied to prioritize causal genes. Single-cell eQTL-based MR across 14 immune lineages generated cell-type-specific causal hypotheses, which were validated using human hepatic single-cell RNA-sequencing data (GSE136103). Two-step mediation MR quantified upstream epigenetic and downstream metabolic mechanisms. A high-fat diet (HFD)-induced murine model provided organismal validation. **Results:** Multi-layered MR nominated *PLXND1* as a robust causal driver of MASLD. Single-cell eQTL-based MR revealed a functional dichotomy: *PLXND1* upregulation in CD8^+^ effector memory T-cells decreased MASLD risk (OR = 0.486, 95% CI: 0.290–0.813, *p* = 0.006), whereas upregulation in natural killer cells (OR = 1.567, 95% CI: 1.337–1.837, *p* < 0.001), non-classical monocytes, and dendritic cells increased risk. Human hepatic single-cell transcriptomics confirmed that *PLXND1* marks an anti-fibrotic, IFNG-high CD8^+^ T subset and a pro-inflammatory lipid-associated macrophage (LAM) population. Mediation MR identified DNA methylation at cg26767922 and cg08471739 as protective mediators acting predominantly via *PLXND1* downregulation (92.39% and 64.50% mediation, respectively), and linked *PLXND1* to six circulating metabolites. HFD mice showed significant hepatic *PLXND1* upregulation. **Conclusions:** *PLXND1* functions as a lineage-dependent molecular switch in MASLD, validated across genetic, epigenetic, metabolic, and single-cell dimensions. These findings caution against systemic *PLXND1* blockade and support precision therapeutic strategies targeting hepatic innate immune cells.

## 1. Introduction

Metabolic dysfunction-associated steatotic liver disease (MASLD) has emerged as the defining chronic liver disorder of the metabolic era, with a rapidly rising global prevalence that shows no sign of geographical containment [1,2,3,4]. Although the earliest stage presents as seemingly benign hepatic steatosis, the disease can progress relentlessly through fibrosis and cirrhosis to hepatocellular carcinoma, inflicting substantial burdens on healthcare systems, economic productivity, and social welfare [5,6,7,8,9,10]. Despite major strides in understanding the physiological underpinnings of MASLD, effective pharmacological interventions remain scarce, and most approved or late-stage therapies offer only modest benefit [11,12,13,14]. This therapeutic gap reflects the fundamental complexity of MASLD pathogenesis, which cannot be reduced to a single insult; rather, it arises from a dynamic interplay among genetic predisposition, epigenetic programming, metabolic reorganization, and cross-talk between the liver and peripheral immune system [8,15,16,17].

Mendelian randomization (MR) provides a rigorous, quasi-experimental framework to interrogate causal associations in complex diseases [18,19,20]. Driven by increasingly powered genome-wide association studies and the integration of multi-omics datasets—including expression (eQTL), protein (pQTL), and epigenetic quantitative trait loci—MR is uniquely positioned to prioritize mechanistic biomarkers and therapeutic targets [21,22]. However, current MR investigations into MASLD remain highly fragmented, typically analyzing individual molecular layers—such as gene expressions, metabolites, or immune profiles—in isolation. This siloed approach obscures how disease risk propagates across multi-omic strata [23,24,25] and fails to identify the interconnected regulatory hubs that orchestrate MASLD pathogenesis [26,27].

To address these critical limitations, we developed a genetically informed, multi-scale analytical framework that sequentially links population-level causality with single-cell phenotypic validation in human MASLD tissue. By integrating multi-layered two-sample MR, summary-data-based MR (SMR), and cross-omics mediation analyses with high-resolution single-cell eQTL data and human hepatic single-cell RNA-sequencing, we sought to map the causal architecture of MASLD from epigenetic regulation through metabolic remodeling to immune microenvironmental dysfunction [18,28,29]. This approach enabled us to nominate *PLXND1*—a semaphorin receptor with lineage-restricted immune expression and established roles in immune cell trafficking and endothelial remodeling [30]—as a causal driver and to predict and directly validate its striking, cell-type-specific functional dichotomy within the steatotic liver.

Our study moves beyond descriptive association to establish a coherent, cross-omics causative model of MASLD pathogenesis. By closing the loop between genetic prediction and cellular phenotype, we provide a mechanistic blueprint for precision therapeutic strategies that target specific hepatic immune compartments rather than pursuing systemic, blunt inhibition, thereby offering a more rational path toward clinical translation.

## 2. Materials and Methods

### 2.1. eQTL Dataset

The information about local expression quantitative trait loci was based on whole-blood samples of the eQTLGen repository (https://www.eqtlgen.org/cis-eqtls.html, accessed on 10 October 2025) [31]. In order to identify strong genetic proxies, we used tight inclusion criteria, namely, genome-wide significance cut-offs (*p* < 5 × 10^−8^) and linkage disequilibrium pruning (*r*^2^ < 0.1) within a 10,000-kb range. Such an intensive screening finally resulted in finding 15,695 unique loci with adequate local regulatory variants.

### 2.2. pQTL Dataset

The unadjusted summary-level data on pQTL were downloaded from the deCODE repository (https://www.decode.com/summarydata/, accessed on 10 October 2025) [32] and involved 4907 proteomic traits measured among an Icelandic cohort. Cis-acting variants were selected through imposing tight limits in terms of geography: the candidate single-nucleotide polymorphisms (SNPs) had to be located on the same chromosome and had to lie wholly inside a 1-megabase region that enclosed the target gene sequence. Such a geographical limitation identified 4706 cis-oriented assemblies of interest. In order to obtain stringent genetic tools, independent variants were obtained using a strict genome-wide significance threshold (*p* < 5 × 10^−8^) and linkage disequilibrium clumping (distance cutoff = 10,000 kb; *r*^2^ < 0.1). After such quality control steps, there were 1615 strong groups of genetic proxies still accessible for further analysis.

### 2.3. Outcome Dataset

The summary statistics of MASLD were released by the FinnGen consortium (release R12; https://www.finngen.fi/en, accessed on 10 October 2025), consisting of 3504 MASLD patients and 496,844 controls of European ancestry. The large amount of data combines genetic and clinical information, enabling a detailed study of the genetic relationships in MASLD.

### 2.4. Single-Cell eQTL Dataset (sc-eQTL)

The transcriptomic profiles of individual cells were collected using the OneK1K resource (https://onek1k.org/, accessed on 11 December 2025) [33] which is a large library containing about 1.27 million peripheral blood mononuclear cells (PBMCs) sampled in 982 different individuals. We used publicly available, annotated transcriptomic profiles of 14 different immune cell subsets to map the expression patterns of the target gene. This fine-grained method made it possible to identify its distribution in certain immune populations and examine its role in the development of MASLD pathogenesis at the level of a single cell.

### 2.5. Single-Cell RNA-Sequencing Analysis of Human Hepatic Tissues

Publicly available single-cell RNA-sequencing data from human normal and MASLD liver samples were retrieved from the Gene Expression Omnibus (GEO) database under accession number GSE136103. The Seurat package in R was employed for quality control, normalization, dimensionality reduction, clustering, and visualization. For T-cell subclustering, all T-cells were extracted and re-clustered, followed by annotation into CD4^+^ and CD8^+^ subsets using lineage-specific markers. Differential gene expression analysis was performed using the Wilcoxon rank-sum test with Bonferroni correction. Gene Ontology (GO) biological process enrichment and Kyoto Encyclopedia of Genes and Genomes (KEGG) pathway analysis were conducted using the clusterProfiler package (version 4.10.0). For macrophage analysis, cells were stratified into *PLXND1*-positive and *PLXND1*-negative populations, and differential expression and functional enrichment analyses were performed accordingly.

### 2.6. Two-Sample Mendelian Randomization Analysis

We have applied MR models through the TwoSampleMR computational package (v.0.6.13) to interrogate the causal mechanisms underlying MASLD. Our predictive factors were genetic architectures that defined cis-eQTL and cis-pQTL expression, which we compared with the disease phenotype. The protection against weak-instrument distortion required eliminating polymorphisms with F-values less than 10. The main statistics model used in our case was inverse variance-weighted (IVW) protocols, which led to the exclusion of all the traits with low variant counts to be able to apply strict IVW protocols. Further validation measures included MR-Egger intercepts to identify pleiotropic confounders, Cochran’s Q equations to measure variant dispersion and systematic leave-one-out permutations to ensure overall inferential resilience.

### 2.7. Summary-Data-Based Mendelian Randomization (SMR) Analysis

In order to explore possible causal structures between particular transcriptomic or proteomic profiles and MASLD etiology, we have implemented summary-data-based Mendelian randomisation frameworks based on genome-wide and quantitative-trait-locus summary statistics. The Heterogeneity in Dependent Instruments (HEIDI) protocol was incorporated in our pipeline in order to ensure that true pleiotropy could be distinguished in a definitive manner as opposed to simple genetic confounding. The statistical analysis is implemented with a null hypothesis of no horizontal pleiotropic effect; therefore, maintaining the null implies that every observed molecular association is due to a single common polymorphism rather than separate but tightly linked loci that are in linkage disequilibrium. Computational implementation was based upon the SMR suite release 1.3.1 (https://yanglab.westlake.edu.cn/software/smr/#Overview, accessed on 10 October 2025), and standard operating parameters were used.

### 2.8. Mediation Analysis

#### 2.8.1. Upstream Analysis

We used a two-stage MR mediation analysis in an attempt to establish whether epigenetic modifications can regulate transcriptional outputs which eventually lead to the development of MASLD. mQTL statistics, available through the GoDMC consortium registry (http://www.GoDMC.org.uk/, accessed on 11 December 2025) [34], were taken as basic exposures. Instrumental variants were filtered through rigorous criteria, including keeping only those with genome-wide significance (*p* < 5 × 10^−8^) and rigid independence conditions (*r*^2^ < 0.1 in a 10,000-kb genome region). Our analysis pipeline measured three different causal measures: the overall effect of methylation state on the liver condition (βall), the epigenetic effect that determines particular transcript values (β1), and the downstream effect of these RNA transcripts that influence the disease predisposition (β2). We then calculated the indirect mediated pathway by multiplying these intermediate pathways (β12 = β1 × β2) and defined the partial epigenetic effect using the fraction (β12/βall) × 100%.

#### 2.8.2. Downstream Analysis

In order to decode other physiological processes, we have used a two-step MR approach that determines if systemic circulating intermediates can affect the effects of target transcripts on hepatic steatosis. The summary statistics involving 1400 different blood-borne biochemicals were obtained through the official GWAS Catalog database (accession numbers GCST90199621-GCST90201020; https://www.ebi.ac.uk/gwas/, accessed on 11 December 2025). Instrumental variable extraction parameters were exactly based on our previous set filtration criteria. Three important causal vectors were measured: the overall clinical effect which connects transcript amounts with disease phenotype (βall), the initial vector that relates RNA abundance with circulating metabolic substances (β1), and final vector that relates these systemic biomarkers with the development of pathology (β2). The indirect mediated pathways (β12 = β1 × β2) and their respective fractional effects were mathematically calculated by using the same formulaic pipeline described above.

### 2.9. MASLD Mouse Model

Wild-type C57BL/6 male mice (8 weeks old; 20–22 g) were obtained through Shaanxi Yike Medical Biotechnology Co., Ltd (Xi’an, Shaanxi, China). The mice were kept on standard laboratory settings (20–25 °C, 30–50 percent relative humidity, and 12 h of light and darkness). After a habituation period (of seven days), we randomly allocated the murine models on standard laboratory chow (ND) or high-fat diet (HFD; containing 60% of calories as lipids). Each in vivo protocol strictly complied with the ethical requirements set by the Air Force Medical University regulation committee on animal welfare.

### 2.10. Histological Staining of Murine Liver Tissues

Following 12 weeks of dietary intervention, liver tissues from both normal chow and HFD groups were harvested, fixed in 4% paraformaldehyde, and embedded in paraffin. For hematoxylin and eosin (H&E) staining, 5-μm sections were deparaffinized, rehydrated, and stained with hematoxylin and eosin to evaluate general hepatic architecture, steatosis, and hepatocyte ballooning degeneration. For Oil Red O staining, frozen liver sections (8 μm) were fixed in 4% paraformaldehyde, rinsed with distilled water, and stained with 0.5% Oil Red O working solution for 15 min to visualize neutral lipid accumulation. Sections were then counterstained with hematoxylin and examined under a light microscope. For Masson’s trichrome staining, paraffin-embedded liver sections were deparaffinized and rehydrated, followed by staining with Weigert’s iron hematoxylin, Biebrich scarlet-acid fuchsin, and aniline blue to assess collagen deposition and early fibrotic changes. All histological images were captured under a light microscope (Leica Microsystems, Wetzlar, Germany) and analyzed by investigators blinded to the experimental groups.

### 2.11. Quantitative Real-Time PCR (qRT-PCR)

Total RNA was isolated with TRIzol reagent (Invitrogen, Carlsbad, CA, USA) as per the recommendations of the manufacturer. Reverse transcription of RNA into cDNA was performed with Evo M-MLV RT Premix (Accurate Biology, Changsha, China).

SYBR Green Premix Pro Taq HS (Accurate Biology) was used as the reagent in qRT-PCR and the reaction was conducted on a Bio-Rad CFX Maestro 2.2 system (Bio-Rad, Hercules, CA, USA). The internal control was β-actin.

Primer sequences were as follows:

*PLXND1* forward: 5′-CGCAACCGTAGCCTAGAAGAC-3′

*PLXND1* reverse: 5′-GGTTAAGGTCGAAGGTGAAGAG-3′

*β-actin* forward: 5′-GTGACGTTGACATCCGTAAAGA-3′

*β-actin* reverse: 5′-GCCGGACTCATCGTACTCC-3′

### 2.12. Western Blot

The liver samples were homogenized with RIPA extraction reagent (Thermo Fisher Scientific, Waltham, MA, USA, Cat# 89900). Following this, total peptide yields were measured by bicinchoninic acid colorimetry. Electrophoretic resolution of polypeptide fractions was performed on sodium dodecyl sulfate–polyacrylamide gel and electroblotted onto hydrophobic PVDF membranes purchased at Millipore (Billerica, MA, USA). The particular blots were probed overnight with goat-specific anti-*PLXND1*-recognizing immunoglobulins (Abcam, Cambridge, UK; Cat# ab28762) and finally incubated with horseradish peroxidase-conjugated anti-goat conjugates obtained from donkeys (Abcam, Cambridge, UK; Cat# ab97110).

### 2.13. Statistical Analysis

The R environment (v4.4.2; R Core Team) and GraphPad Prism (v10.0) were used to perform computational processing and statistical analyses. We have chosen a bilateral *p*-value of less than 0.05 as the threshold of statistical significance in all inferential tests.

## 3. Results

### 3.1. Multi-Layered MR Identifies PLXND1 as a Causal Driver of MASLD

**Figure 1:**
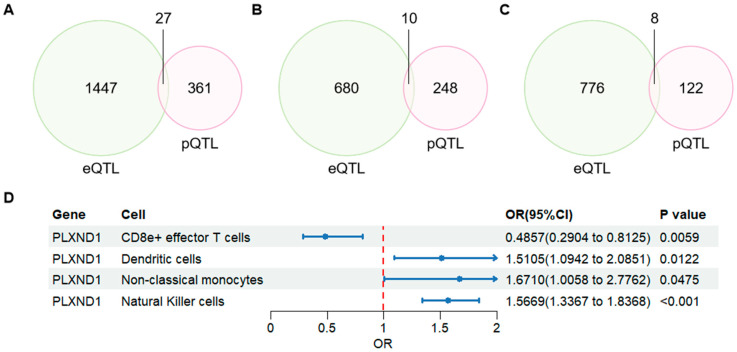
Multi-layered MR identifies PLXND1 as a causal driver of MASLD. (**A**–**C**) Venn diagram showing the overlap of genes significantly associated with MASLD at the transcriptomic (eQTL) and proteomic (pQTL) levels. (**D**) Forest plot of single-cell eQTL-based MR estimates showing the cell-type-specific causal effects of genetically predicted PLXND1 expression on MASLD susceptibility across peripheral blood immune subpopulations. Displayed are odds ratios (ORs) and their 95% confidence intervals (CIs); arrows indicate CIs exceeding the x-axis limits.

We have conducted a two-sample MR analysis to explore the possible causal relationships between gene expression and MASLD in the context of transcriptomic and proteomic levels. The exposure variables were cis-acting quantitative trait loci, such as cis-expression quantitative trait loci (cis-eQTL) and cis-protein quantitative trait loci (cis-pQTL), and the outcome was summary-level data of a MASLD genome-wide association study (GWAS). All SNPs included in the instrumental variable selection had statistics of over 10, so it is highly unlikely that weak-instrument bias would occur. The inverse-variance weighted (IVW) was implemented as the main analytic strategy, and the value of *p* less than 0.05 was considered statistically significant. We found 1475 genes at the transcriptional (Supplementary Table S1) and 392 genes at the protein level (Supplementary Table S2) that are significantly and causally related to MASLD. Integration of these findings across layers generated 27 candidate genes with potential causal relevance to MASLD across both regulatory levels (Figure 1A). Additional evaluation of the effect direction showed that 18 of these genes had concordant causal patterns (10 of them were consistently correlated with higher risk of MASLD, and the other 8 with lower risk) (Figure 1B,C).

Evaluation of SMR of the 18 candidate genes indicated that the expression of *PLXND1* (*P_SMR* = 0.0256), GSTZ1 (*P_SMR* = 0.0316) and RPIA (*P_SMR* = 0.0422) was associated with MASLD risk. In order to exclude false linkage disequilibrium, we have used HEIDI testing. All three loci passed this threshold (*P_HEIDI* > 0.05), confirming that direct causality or pleiotropy is the underlying cause of these associations (Table 1). Because *PLXND1* is predominantly expressed in specific leukocyte subsets and has been implicated in immune cell–endothelial crosstalk, its causal effect on MASLD likely operates through cell-type-specific mechanisms that are invisible to bulk-tissue analyses. We therefore decomposed its genetic effect across individual immune lineages using single-cell eQTL-based MR.

### 3.2. Single-Cell eQTL MR Reveals a Functional Dichotomy of PLXND1 Across Immune Lineages

The MASLD immune landscape is also well documented to be patchy, and therefore we need to move beyond tissue analysis to achieve the real picture of the molecular dance taking place. We have measured the genetically estimated expression levels of *PLXND1* in 14 peripheral blood immune subpopulations using single-cell eQTL data with the OneK1K cohort. The findings of the MR revealed the existence of a new reality. Rather than a nonspecific effect, the influence of *PLXND1* on MASLD vulnerability is highly cell specific, working as a polarized rheostat among different immune elements (Figure 1D).

The gene seems to be used by the adaptive immune system as a defensive tool. Genetic fixity of increased *PLXND1* expression exclusively in CD8^+^ effector T-cells (CD8^+^ Tem) means an unexpectedly strong decrease in the risk of MASLD (OR = 0.486, 95% CI = 0.290–0.813, *p* = 0.0059). However, entering the domain of the innate immune system reverses this situation completely, converting the gene into a particular liability. The maximum risk amplification impact occurred between natural killer (NK) cells (OR = 1.567, 95% CI = 1.337–1.837, *p* < 0.001), and an analogous pathogenic signature was also observed in non-classical monocytes and dendritic cells (OR = 1.511, 95% CI = 1.094–2.085, *p* = 0.0122). This dramatic functional dichotomy, protective in the adaptive arm but feeding pathogenesis actively along the innate lineages, places *PLXND1* as a very context-specific, core node controlling the disease architecture.

### 3.3. Human Hepatic Single-Cell Transcriptomics Validate the Cell-Type-Specific Roles of PLXND1

**Figure 2:**
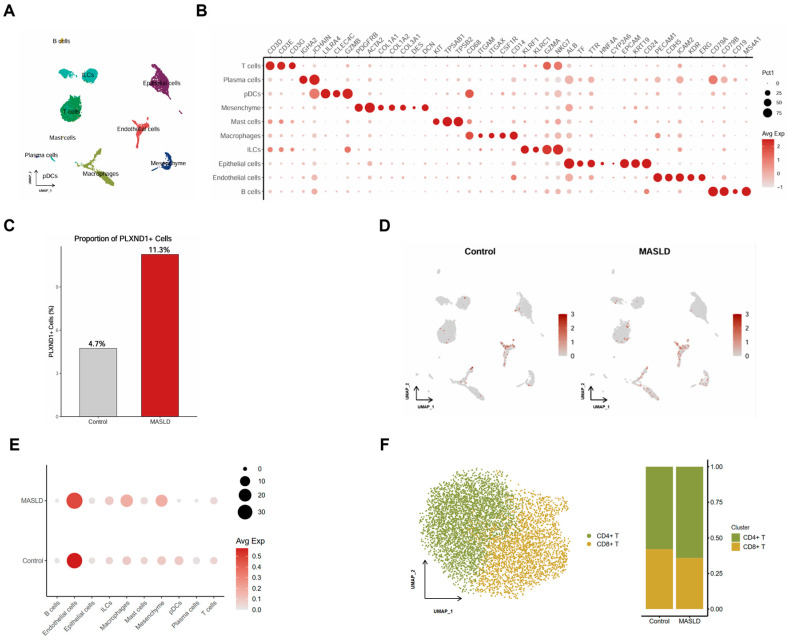
Landscape of PLXND1 expression in the human MASLD liver at single-cell resolution. (**A**) UMAP plot of the GSE136103 dataset showing nine major annotated cell populations. (**B**) Dot plot of canonical marker genes used for cell-type annotation. (**C**) Bar plot quantifying the proportion of PLXND1-expressing cells in control versus MASLD samples. (**D**) Feature plots displaying the spatial distribution of PLXND1 expression across the UMAP in control and MASLD livers. (**E**) Dot plot summarizing PLXND1 expression levels and the percentage of expressing cells across all identified cell types. (**F**) UMAP plot of subclustered T-cells colored by CD4^+^ and CD8^+^ identity and proportional composition in control and MASLD livers; subsets were annotated using canonical lineage markers (CD4, CD8A, CD8B, and FOXP3).

While the sc-eQTL MR predictions are genetically grounded, they derive from peripheral blood and do not reveal the transcriptional programs or disease-associated states that these cells adopt within the steatotic liver itself. To bridge this gap and test whether the predicted functional dichotomy is reflected in human MASLD tissue, we analyzed publicly available single-cell RNA-sequencing data from normal and MASLD liver samples (GSE136103). Uniform manifold approximation and projection (UMAP) resolved nine major cell populations, including T-cells, B cells, macrophages, endothelial cells, and epithelial cells (Figure 2A,B). Comparison of cellular proportions between control and MASLD livers revealed a restructured immune microenvironment in the diseased state, with notable shifts in immune cell representation. Strikingly, the proportion of *PLXND1*-expressing cells was markedly elevated in MASLD livers compared to controls (11.3% versus 4.7%; Figure 2C). Feature plots mapping *PLXND1* expression onto the UMAP space showed that the *PLXND1* signal was most concentrated in endothelial cells and specific immune subsets, with a clear expansion of the *PLXND1*-positive population in MASLD (Figure 2D). Quantification across cell types confirmed that endothelial cells harbored the highest *PLXND1* expression levels, while macrophages and T-cells also displayed detectable and disease-enriched expression (Figure 2E), establishing that *PLXND1*-upregulated cells constitute a genuine cellular component of the human MASLD liver.

**Figure 3:**
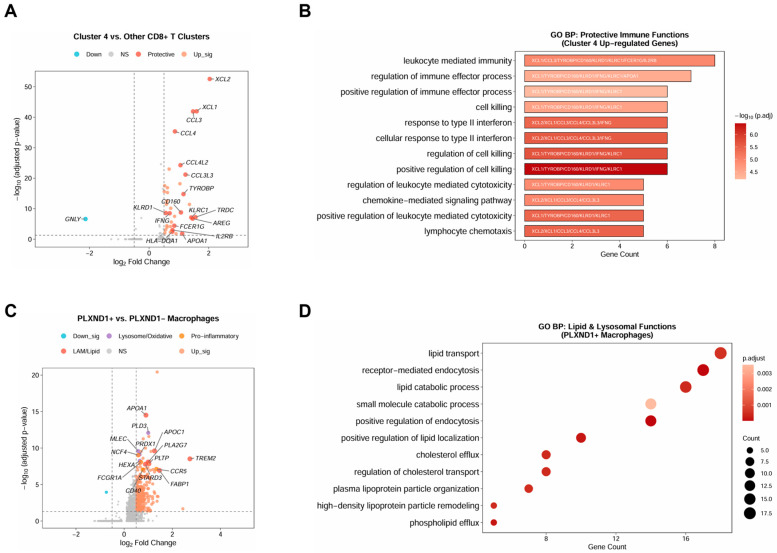
PLXND1 marks functionally dichotomous immune subsets in human MASLD livers. (**A**) Volcano plot of differentially expressed genes comparing PLXND1-high Cluster 4 against all other CD8^+^ T-cell clusters. (**B**) Gene Ontology (GO) biological process enrichment of upregulated genes in Cluster 4. (**C**) Volcano plot of differentially expressed genes between PLXND1-positive and PLXND1-negative macrophages. (**D**) GO biological process enrichment of upregulated genes in PLXND1-positive macrophages. In volcano plots, dashed vertical lines indicate the log_2_ fold-change cutoff, and the dashed horizontal line indicates the adjusted *p*-value significance threshold, such as |log_2_FC| = 0.5 and adjusted *p* = 0.05.

We next dissected the T-cell compartment to interrogate the protective role of *PLXND1* in adaptive immunity. Subclustering of all T-cells distinguished CD4^+^ and CD8^+^ lineages (Figure 2F). Among the CD8^+^ T-cell subsets, Cluster 4 exhibited the highest *PLXND1* expression and was proportionally expanded in MASLD livers. Differential expression analysis comparing Cluster 4 against all other CD8^+^ T-cell clusters revealed a distinct transcriptional signature dominated by cytotoxic effector molecules, including killer lectin-like receptors (KLRC1, KLRD1), the transmembrane receptor CD160, the adaptor TYROBP, and the cytokine IFNG, alongside potent chemoattractants XCL1 and XCL2 (Figure 3A). Gene Ontology biological process enrichment demonstrated significant enrichment in leukocyte-mediated immunity, positive regulation of cell killing, cellular response to type II interferon, and lymphocyte chemotaxis (Figure 3B). These transcriptional features—characterized by high IFNG and cytotoxic molecule expression—suggest that *PLXND1* marks an effector CD8^+^ T-cell subset with anti-fibrotic potential, aligning with the protective causal effect of genetically elevated *PLXND1* in CD8^+^ effector memory T-cells.

In mirror image to the protective adaptive signature, the sc-eQTL MR had predicted that *PLXND1* amplifies MASLD risk along innate immune lineages. To identify the cellular phenotype underlying this pathogenic effect, we compared *PLXND1*-positive versus *PLXND1*-negative macrophages within the same human MASLD dataset. The volcano plot of differentially expressed genes revealed that *PLXND1*-positive macrophages were robustly characterized by the upregulation of established lipid-associated macrophage (LAM) markers, including *TREM2, APOC1, APOA1, PLA2G7, PLTP,* and *FABP1* (Figure 3C). These cells also showed elevated expression of pro-inflammatory mediators such as FCGR1A and CCR5. Gene Ontology enrichment analysis underscored a strong metabolic reprogramming phenotype, with significant enrichment in lipid transport, receptor-mediated endocytosis, lipid catabolic process, cholesterol efflux, and plasma lipoprotein particle remodeling (Figure 3D). Fisher’s exact test indicated that *PLXND1*-positive macrophages were significantly overrepresented in MASLD livers compared to controls (19.1% versus 5.9%, *p* < 0.001). Collectively, these findings establish that *PLXND1* in macrophages marks a metabolically active, pro-inflammatory LAM subset that accumulates in the MASLD liver, providing a plausible cellular mechanism for the risk-enhancing causal effect of *PLXND1* in innate immune lineages.

Taken together, these human hepatic single-cell transcriptomic data validate the functional dichotomy predicted by genetic MR: *PLXND1* is associated with protective cytotoxic effector programs in CD8^+^ T-cells, whereas it defines a pathogenic lipid-associated macrophage phenotype in the innate immune compartment. This context-dependent behavior positions *PLXND1* not as a uniform disease driver or suppressor, but as a cell-type-specific molecular switch in the MASLD immune microenvironment.

### 3.4. Cross-Omics Mediation Links PLXND1 to Epigenetic and Metabolic Remodeling

**Figure 4:**
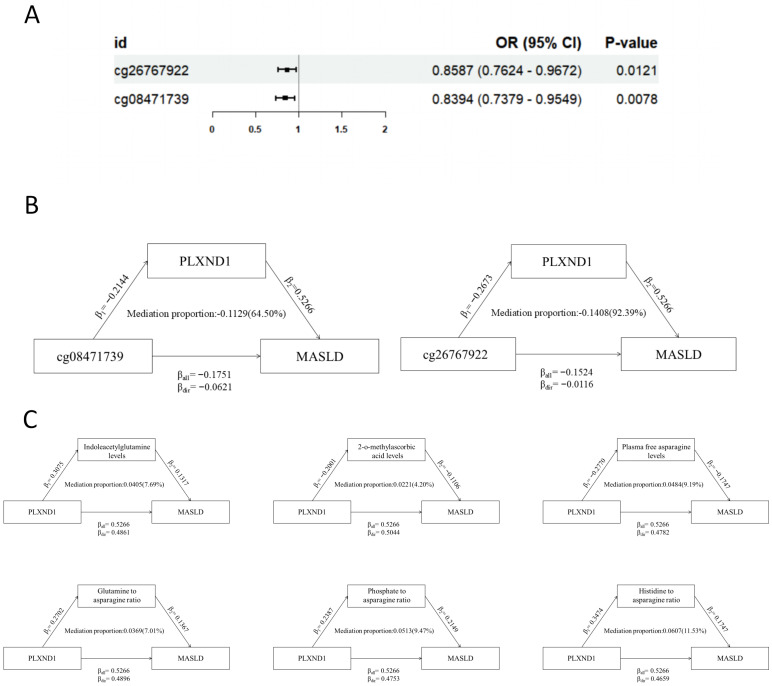
Cross-omics mediation links PLXND1 to upstream epigenetic programming and downstream metabolic remodeling. (**A**) Forest plot depicting the causal effects of DNA methylation quantitative trait loci (mQTLs) at CpG sites cg26767922 and cg08471739 on MASLD risk. (**B**) Path diagrams illustrating the two-step mediation MR framework for cg26767922 and cg08471739, quantifying the proportion of the total protective effect mediated through PLXND1 downregulation. (**C**) Directed acyclic graphs showing the mediation of PLXND1 expression on MASLD risk through six distinct circulating blood metabolites.

In addition to its cell-type-specific immunological roles, the causal impact of *PLXND1* on MASLD is shaped by upstream regulatory mechanisms and downstream systemic effectors. We therefore turned to cross-omics mediation analyses to quantify how epigenetic modifications and circulating metabolites propagate disease risk through *PLXND1*. The data revealed a signal that was surprisingly clear: methylation of two separate CpG sites, cg08471739 and cg26767922 lead to a highly significant causal switch in the disease susceptibility (Figure 4A). And, more specifically, how do these epigenetic switches work? It was quite obvious that we should ask ourselves if they can directly control the outcome of MASLD by promoting or inhibiting the expression of targeted genes. In accordance with this causal chain (β1), as expected, the two loci are significantly influencing the expression of *PLXND1* (Table 2). To examine the cascade wiring in more detail, we implemented a mediation MR model. The readouts were really shocking. The shielding of MASLD of cg26767922 is not just an indirect by-product of some other process—no less than 92.39 percent of it is caused by structural processes, in particular, by the specific downregulation of *PLXND1* (Figure 4B). The subsequent locus, cg08471739, performs in the same way though perhaps somewhat weaker as inhibition of *PLXND1* accounts of about 64.50 percent of its total protective impact (Figure 4B).

The massive *PLXND1* effect on MASLD risk means that a larger downstream metabolic signature is present. The physiological cascade caused by this genetic switch necessitated expanding our mediation MR model to actively seek through the circulating effector molecules. Our first step involved identifying 69 different blood metabolites which may be associated with the presence of MASLD due to their demonstrable relationship (Supplementary Table S3). The initial challenge was to locate *PLXND1* in the correct position within this metabolic hierarchy. With *PLXND1* eQTL as our primary exposure, we utilized it to identify whether the gene is actually an upstream regulator that causes these specific alterations. This readout was exceptionally narrow. It turned out that the expression of *PLXND1* directly controls the serum levels of just six different metabolites (Table 3). Equipped with this surprising clear molecular bridge, we performed a final mediation MR experiment measuring the indirect effects passing via this novel pathway (Figure 4C).

### 3.5. Hepatic PLXND1 Is Upregulated in HFD-Induced MASLD Mice

**Figure 5:**
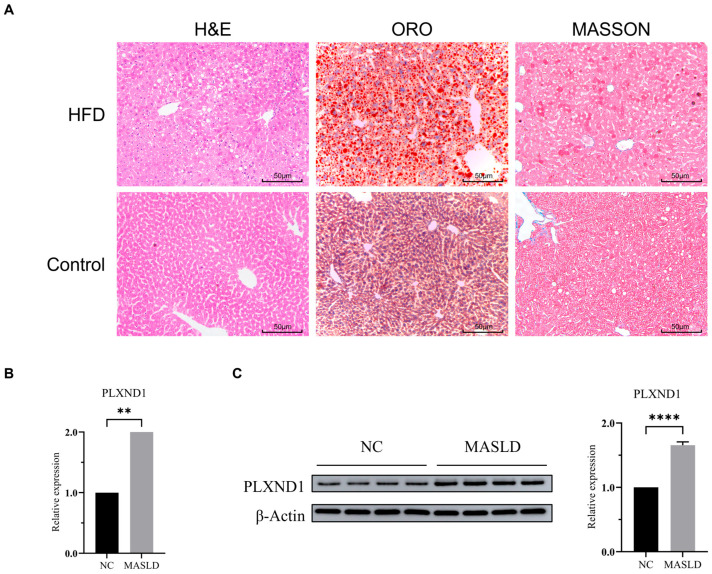
Hepatic PLXND1 is upregulated in HFD-induced MASLD mice. (**A**) Representative histological images of liver sections from normal chow (NC) and high-fat diet (HFD) mice, including hematoxylin and eosin (H&E) staining, Oil Red O staining, and Masson’s trichrome staining. (**B**) Quantitative real-time PCR analysis of hepatic PLXND1 transcript levels. (**C**) Representative immunoblot (**left**) and corresponding densitometric quantification (**right**) of hepatic PLXND1 protein expression normalized to β-Actin. Statistical significance is indicated by asterisks: ** *p* < 0.01; **** *p* < 0.0001.

While the multi-omics computational analyses above establish a coherent causal chain from epigenetic perturbation to metabolic remodeling, they remain inferential; experimental validation is required to confirm that *PLXND1* is genuinely upregulated in the steatotic liver. To establish a relevant in vivo disease context for the computational and human tissue findings, we utilized a HFD-induced murine model of MASLD. Male C57BL/6 mice were fed either a normal chow diet or an HFD (60% kcal from fat) for 12 weeks. Histological evaluation of liver sections confirmed successful metabolic injury induction in the HFD group. Hematoxylin and eosin (H&E) staining revealed pronounced hepatocyte ballooning degeneration and steatosis in HFD-fed mice compared to the normal hepatic architecture observed in NC controls (Figure 5A). Oil Red O staining demonstrated markedly increased lipid accumulation within hepatocytes of the HFD group (Figure 5A). Furthermore, Masson’s trichrome staining indicated elevated collagen deposition, signifying the presence of early fibrotic changes in steatotic livers (Figure 5A).

Having confirmed the MASLD phenotype at the tissue level, we next assessed hepatic *PLXND1* expression. Quantitative real-time PCR showed a significant increase in *PLXND1* transcript abundance in the livers of HFD-fed mice relative to NC-fed controls (Figure 5B). Immunoblot analysis corroborated this finding at the protein level, with densitometric quantification revealing a robust elevation of hepatic *PLXND1* in the MASLD cohort (Figure 5C). Collectively, these multi-level data demonstrate that *PLXND1* is significantly upregulated in the steatotic liver, providing experimental validation that the gene is not merely a statistical artifact of genetic inference but is actively engaged in the in vivo pathogenesis of MASLD.

## 4. Discussion

Current MR studies of MASLD have made important strides in identifying causal risk factors and prioritizing therapeutic targets [18,35,36]. Large-scale genome-wide association studies coupled with expression and protein quantitative trait loci have nominated numerous genes and pathways that influence disease susceptibility [26,37]. However, the field remains largely fragmented as most investigations analyze transcriptomic, proteomic, or metabolomic layers in isolation while the causal propagation of risk across these strata is rarely dissected in an integrated manner [38,39]. Consequently, while individual molecular associations have accumulated, the interconnected regulatory hubs that truly orchestrate MASLD pathogenesis have remained elusive [27,40,41,42].

In the present study, we focused on *PLXND1* as a central node that bridges upstream epigenetic regulation, downstream metabolic reprogramming, and cell-type-specific immune dysfunction. Our multi-layered MR framework first established *PLXND1* as a robust causal driver of MASLD at both the transcriptomic and proteomic levels. Mediation analyses then demonstrated that DNA methylation at cg26767922 and cg08471739 exerts protective effects primarily through the transcriptional downregulation of *PLXND1*, and that *PLXND1* propagates disease risk via six circulating blood metabolites. Most importantly, single-cell eQTL-based MR revealed a striking functional dichotomy: genetically elevated *PLXND1* expression was protective in CD8^+^ effector memory T-cells but pathogenic in natural killer cells, non-classical monocytes, and dendritic cells. Human hepatic single-cell transcriptomics directly validated these predictions by showing that *PLXND1* marks a cytotoxic, IFNG-high CD8^+^ T subset with anti-fibrotic potential, while in macrophages it defines a disease-enriched, pro-inflammatory LAM subset. Finally, in vivo validation in high-fat diet-induced MASLD mice confirmed significant hepatic upregulation of *PLXND1* in steatotic livers. Together, these findings trace a continuous causal chain from epigenetic perturbation through metabolic remodeling to immunometabolic dysfunction.

The central advance of our work lies in its deliberate bridging of population-level genetic causality with tissue-level cellular phenotyping. Previous multi-omics studies in MASLD have successfully mapped genetic, transcriptomic, and proteomic associations in parallel [26,43,44,45], yet they have largely remained descriptive or correlative, without establishing how a single molecular hub drives distinct cellular behaviors in the diseased liver [41,46]. Similarly, single-cell transcriptomic atlases of MASLD have richly documented hepatic immune heterogeneity [47], but because they lack genetic instrumentation, they cannot distinguish whether observed gene-expression patterns are causally implicated in disease or merely represent downstream consequences of metabolic injury [48,49]. The integration of single-cell eQTL data with MR has recently emerged as a powerful strategy to resolve cell-type-specific causal effects in autoimmune and infectious diseases [50], but its application to MASLD—particularly with direct validation in human steatotic livers—has, to our knowledge, not been attempted. By genetically predicting a cell-type-specific functional dichotomy and then corroborating it in human hepatic single-cell data, we close a critical gap between statistical causality and biological mechanism.

Several specific innovations distinguish this study from existing studies. First, rather than treating epigenetics, transcriptomics, metabolomics, and immunology as parallel but disconnected stories, we modeled them as sequential links in a unified causal chain. This enabled us to quantify, for example, that 92.39% of the protective effect of cg26767922 operates through *PLXND1* downregulation, and to trace how *PLXND1* upregulation reshapes circulating metabolites that in turn influence MASLD risk. Second, the identification of *PLXND1* as a lineage-dependent molecular switch—protective in adaptive cytotoxic T-cells yet pathogenic in innate LAMs—challenges binary classifications of genes as uniformly beneficial or harmful. This context-dependent behavior explains why bulk-tissue transcriptomic studies of MASLD have often reported contradictory immune signatures: opposing effects across cell populations become attenuated or erased when tissue is homogenized. Third, our findings carry direct translational implications. Systemic pharmacological blockade of *PLXND1* would simultaneously eliminate the protective cytotoxic CD8^+^ T-cell response and suppress the pathogenic LAM phenotype. Our data therefore support a shift toward cell-type-specific precision strategies—such as macrophage-targeted nanocarriers or liver-homing biologics—that selectively modulate *PLXND1* in hepatic innate immune cells rather than bluntly inhibiting it throughout the organism.

The precise downstream mechanisms through which PLXND1 exerts its lineage-specific effects remain to be defined. As a direct receptor for class 3 semaphorins—most notably Sema3E—PLXND1 canonically signals through its intracellular GAP domain to suppress R-Ras/M-Ras activity, thereby regulating cytoskeletal remodeling, integrin-dependent adhesion, directional migration, and vascular morphogenesis [51,52]. Whether analogous semaphorin-guided mechanisms operate in hepatic innate immunity—for instance, directing PLXND1-expressing LAMs toward lipid-laden hepatocytes—remains speculative, and the downstream effector cascades operative in this context are unknown. The present single-cell transcriptomic analysis independently revealed enrichment of PLXND1^+^ macrophages in lipid transport, PPAR signaling, and receptor-mediated endocytosis gene sets, providing transcriptional evidence for a functional link between PLXND1 expression and the lipid-sensing and efferocytosis programs characteristic of LAMs in steatotic liver disease [53,54,55]. In CD8^+^ effector memory T-cells, PLXND1 has been implicated in modulating cell motility [56], and it is conceivable that semaphorin-guided positioning may contribute to cytotoxic T-cell trafficking within fibrotic liver tissue; however, the proposed coordination of XCL1/XCL2 secretion through PLXND1 signaling remains entirely hypothetical and requires experimental validation [57,58]. Collectively, these observations suggest that semaphorin-receptor signaling, immune cell positioning, and transcriptional lipid-metabolic programming may represent candidate mechanisms linking PLXND1 to MASLD-associated immune responses. Defining the precise context-dependent cascades will require receptor–ligand interaction profiling, phosphoproteomic characterization of PLXND1 signaling complexes in primary hepatic immune cells, and cell-type-resolved functional assays.

Several limitations warrant consideration. First, although MR-Egger intercept tests, Cochran’s Q assessments, and leave-one-out permutations were performed, residual horizontal pleiotropy cannot be entirely excluded. The concordant causal directionality across both eQTL- and pQTL-derived instruments, together with SMR-HEIDI co-localization confirming a shared causal variant rather than linkage disequilibrium artifacts, nonetheless supports the robustness of our principal estimates. Second, the underlying GWAS statistics derive predominantly from European-ancestry cohorts—FinnGen R12, eQTLGen, and deCODE—limiting generalizability to non-European populations, including East Asian groups in whom MASLD genetic architecture and environmental determinants may differ substantially. Third, single-cell analyses relied on a single publicly available dataset (GSE136103); the PLXND1-expressing LAM and CD8^+^ T-cell phenotypes described herein require corroboration in independent datasets with matched clinical and histological annotation. Fourth, while the HFD murine model confirms PLXND1 upregulation in steatotic liver at both transcript and protein levels, the absence of cell-type-specific conditional knockout experiments leaves the directional causal contributions of PLXND1 within individual hepatic immune compartments formally unestablished.

These limitations define a clear path forward. Replication in large-scale multi-ethnic resources—including Biobank Japan and the China Kadoorie Biobank—will establish cross-population generalizability, while independent scRNA-seq profiling of geographically diverse MASLD biopsies will validate the immune phenotypes identified here. Myeloid-specific and cytotoxic T-cell-specific conditional knockout models will permit direct in vivo interrogation of lineage-dependent contributions to steatohepatitis and fibrosis; parallel dissection of Sema3E–PLXND1–RhoA/PI3K signaling in sorted hepatic immune populations will identify the molecular nodes most amenable to intervention. Macrophage-targeted delivery platforms capable of selectively silencing Plxnd1 in hepatic myeloid cells while preserving CD8^+^ T-cell function will then provide the translational bridge between these mechanistic insights and precision therapeutic strategies for MASLD.

In summary, this integrative multi-omics framework establishes a transformative paradigm for understanding MASLD pathogenesis by demonstrating how epigenetic regulation, metabolic rewiring, and immune hyperresponsiveness converge through a single molecular hub. The cell type-dependent duality of *PLXND1* underscores that therapeutic modulation of such axes must account for cellular context, highlighting the necessity of precision medicine approaches in the MASLD field.

## 5. Conclusions

This study identifies *PLXND1* as a robust, cell-type-specific causal driver of MASLD. By integrating multi-layered MR with human hepatic single-cell transcriptomics and in vivo validation, we trace a continuous causal chain from DNA methylation through gene expression and metabolic remodeling to divergent adaptive and innate immune phenotypes in the steatotic liver. These findings establish *PLXND1* as a lineage-dependent molecular switch and provide a mechanistic foundation for precision therapeutic strategies that selectively target hepatic innate immune cells.

## Figures and Tables

**Table 1:** Proteomic Discoveries Derived via Summary-Data-Based Mendelian Randomization in MASLD.

Gene	Exposure	Outcome	topSNP	P_SMR	P_HEIDI	nsnp_HEIDI
ENSG00000004399	*PLXND1*	MASLD	rs9866653	0.0256	0.7737	7
ENSG00000100577	GSTZ1	MASLD	rs2363642	0.0316	0.4315	20
ENSG00000153574	RPIA	MASLD	rs10185049	0.0422	0.6663	4
ENSG00000135919	SERPINE2	MASLD	rs13412535	0.0776	0.6226	15
ENSG00000104899	AMH	MASLD	rs4807216	0.1163	0.7914	20
ENSG00000197766	CFD	MASLD	rs71335276	0.2490	0.2778	20
ENSG00000113249	HAVCR1	MASLD	rs2033477	0.4613	0.7131	20

Analytical abbreviations employed throughout this dataset comprise the following: SMR, denoting summary-data-based Mendelian randomization methodologies; HEIDI, representing diagnostic evaluations for heterogeneity in dependent instruments; alongside SNP, which signifies individual single-nucleotide polymorphism markers.

**Table 2:** Mendelian randomization results for key CpG sites significantly associated with *PLXND1* expression.

ID	nSNP	β	se	*p*-Value	Egger_Intercept	Egger_Intercept_se	Egger_Intercept_pval
cg26767922	9	−0.2673	0.0537	<0.001	0.0263	0.0320	0.4378
cg08471739	6	−0.2144	0.0532	<0.001	−0.0191	0.0273	0.5238

**Table 3:** Causal effects of *PLXND1* expression on six blood metabolites.

ID	Reported Trait	nSNP	β	se	*p*-Value
GCST90199817	Indoleacetylglutamine levels	6	0.3075	0.1099	0.0051
GCST90199908	2-o-methylascorbic acid levels	6	−0.2001	0.0926	0.0307
GCST90200452	Plasma free asparagine levels	6	−0.2770	0.0978	0.0046
GCST90200787	Glutamine to asparagine ratio	6	0.2702	0.0969	0.0053
GCST90200866	Phosphate to asparagine ratio	6	0.2387	0.0962	0.0131
GCST90200989	Histidine to asparagine ratio	6	0.3474	0.0982	0.0004

## Data Availability

All data are available in the main text or the Supplementary Materials. The datasets analyzed during the current study are available from the corresponding author upon reasonable request.

## Supplementary Materials

The following supporting information can be downloaded at: https://www.mdpi.com/article/10.3390/metabo16060378/s1, File S1: PLXND1-Mediation Analysis; Table S1: Transcriptome-wide Mendelian randomization results for the causal effects of gene expression on MASLD risk; Table S2: Proteome-wide Mendelian randomization results for the causal effects of circulating protein levels on MASLD risk; Table S3: Mendelian randomization screening of blood metabolites associated with MASLD risk.

## Abbreviations

The following abbreviations are used in this manuscript:

CI	Confidence interval
DEG	Differentially expressed gene
FC	Fold change
GEO	Gene Expression Omnibus
GO	Gene Ontology
GWAS	Genome-wide association study
H&E	Hematoxylin and eosin
HFD	High-fat diet
IVW	Inverse-variance weighted
KEGG	Kyoto Encyclopedia of Genes and Genomes
LAM	Lipid-associated macrophage
MASLD	Metabolic dysfunction-associated steatotic liver disease
mQTL	Methylation quantitative trait locus
MR	Mendelian randomization
NC	Normal chow
NK	Natural killer
OR	Odds ratio
pQTL	Protein quantitative trait locus
qRT-PCR	Quantitative real-time polymerase chain reaction
SMR	Summary-data-based Mendelian randomization
sc-eQTL	Single-cell expression quantitative trait locus
scRNA-seq	Single-cell RNA sequencing
UMAP	Uniform manifold approximation and projection
