# Supplementary figures and images for: Genetically Informed Single-Cell Analysis Reveals PLXND1 as a Cell-Type-Specific Molecular Switch in MASLD
Source: Metabolites. 2026 May 30;16(6):378. doi: 10.3390/metabo16060378 (PMC13303273; doi:10.3390/metabo16060378)

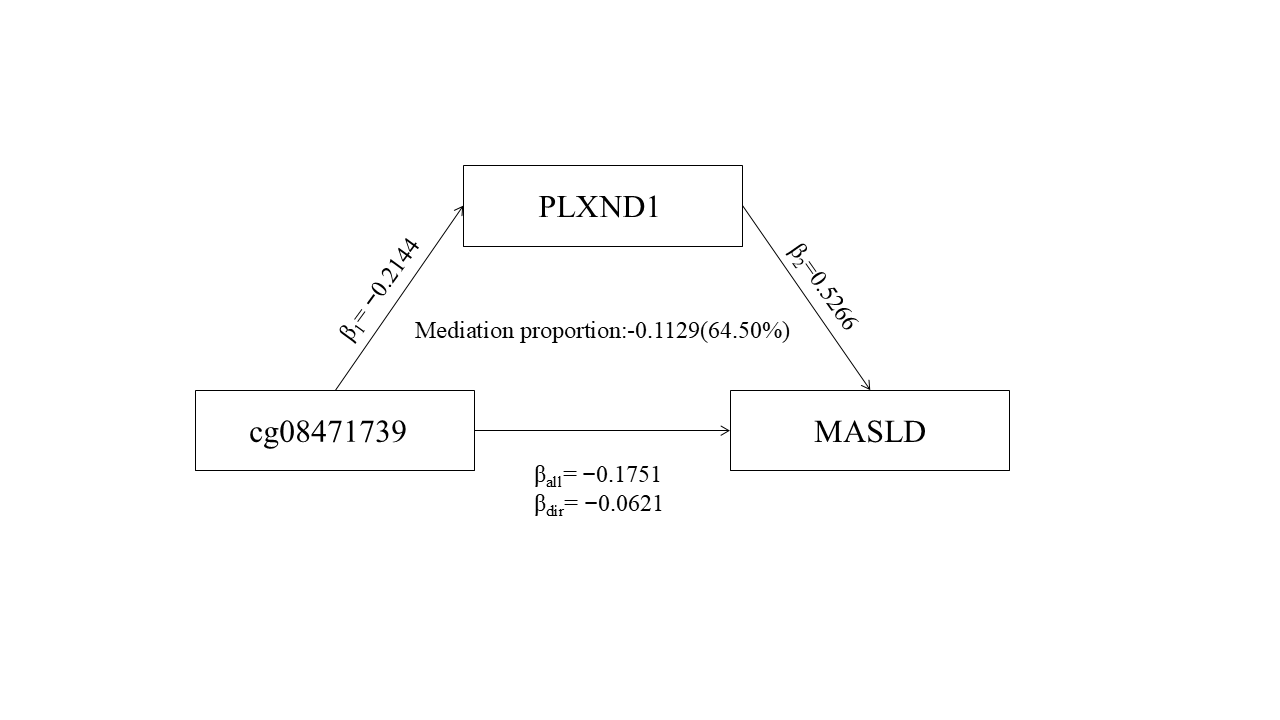

Supplement: Supplementary file 1 [file metabolites-16-00378-s001.zip › File S1. PLXND1-中介效应分析/幻灯片1.TIF]

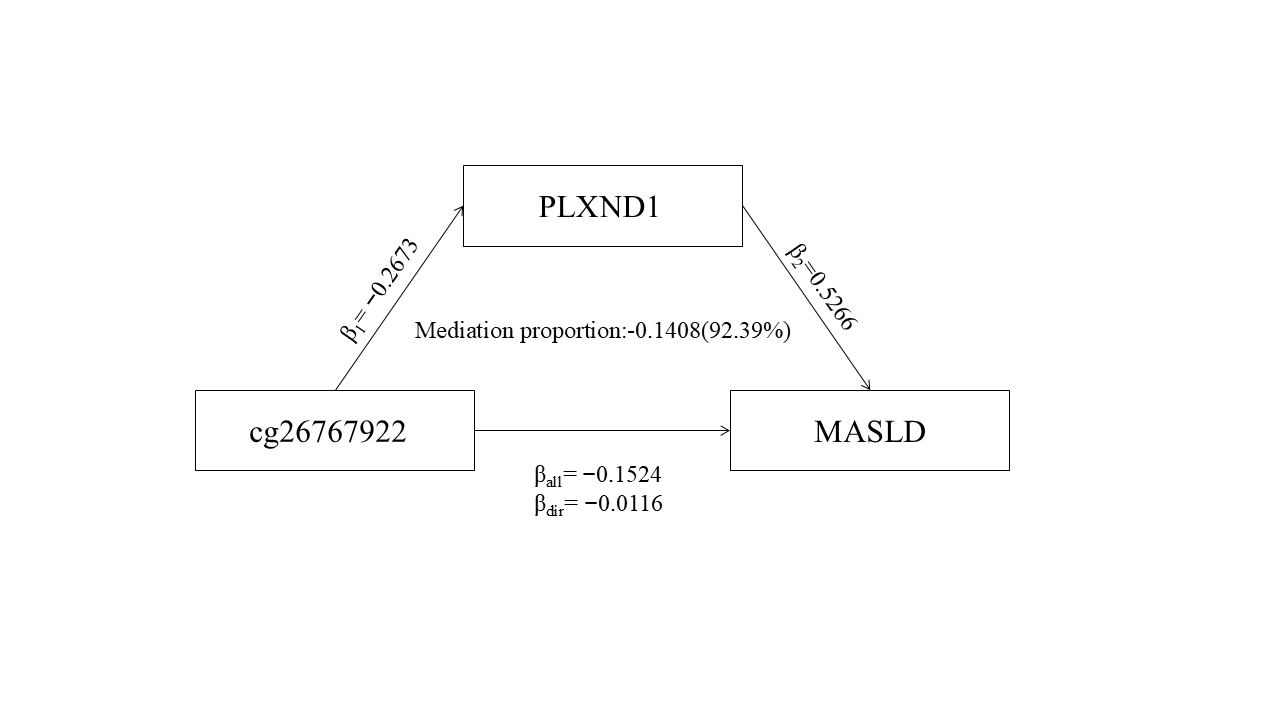

Supplement: Supplementary file 1 [file metabolites-16-00378-s001.zip › File S1. PLXND1-中介效应分析/幻灯片2.TIF]

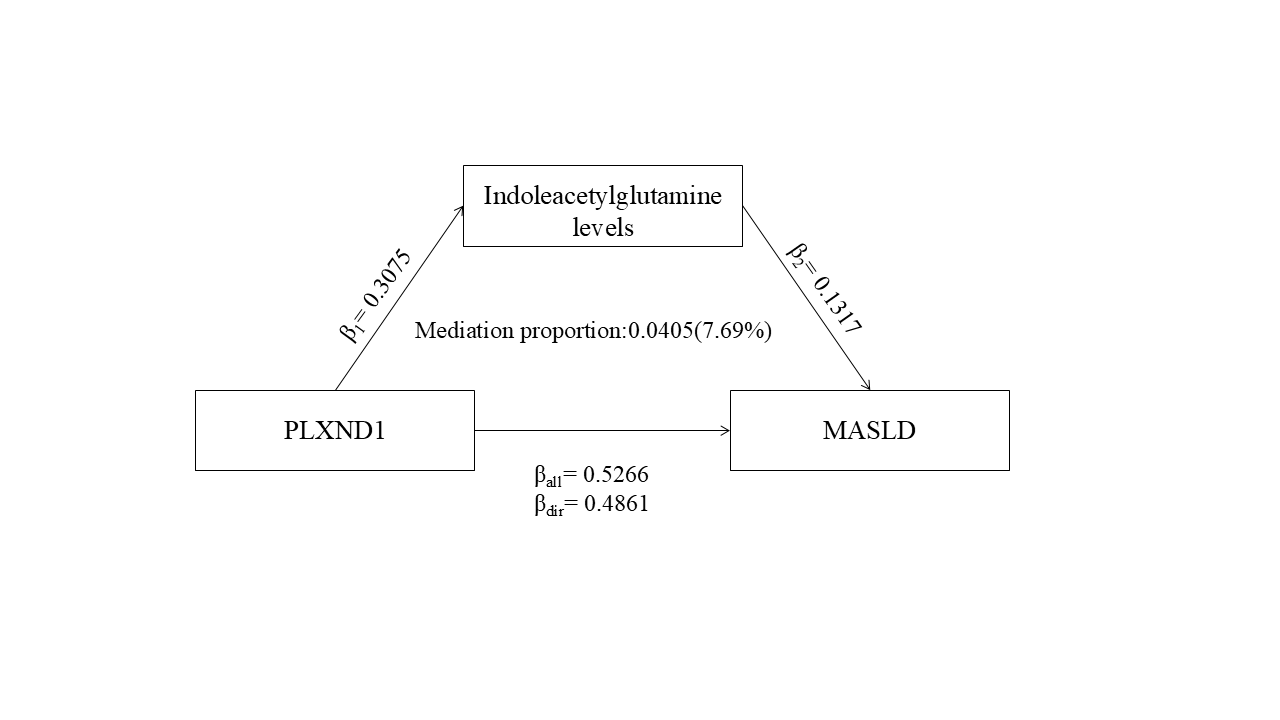

Supplement: Supplementary file 1 [file metabolites-16-00378-s001.zip › File S1. PLXND1-中介效应分析/幻灯片3.TIF]

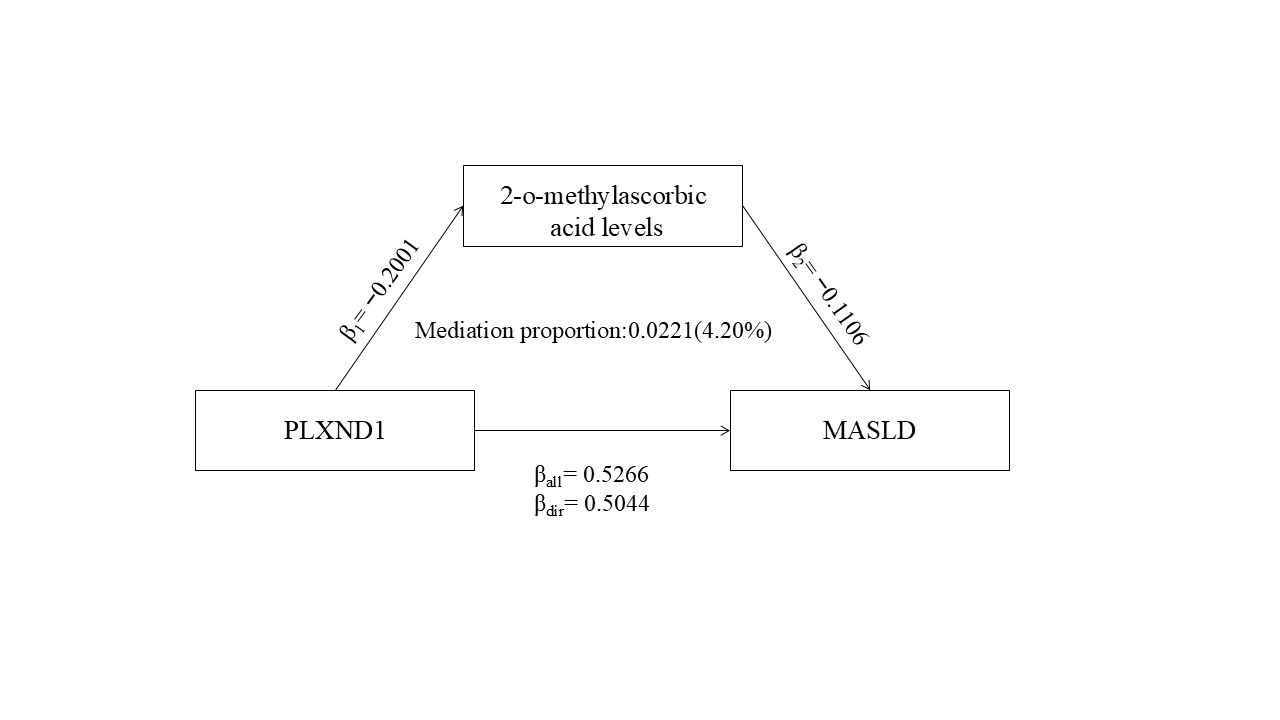

Supplement: Supplementary file 1 [file metabolites-16-00378-s001.zip › File S1. PLXND1-中介效应分析/幻灯片4.TIF]

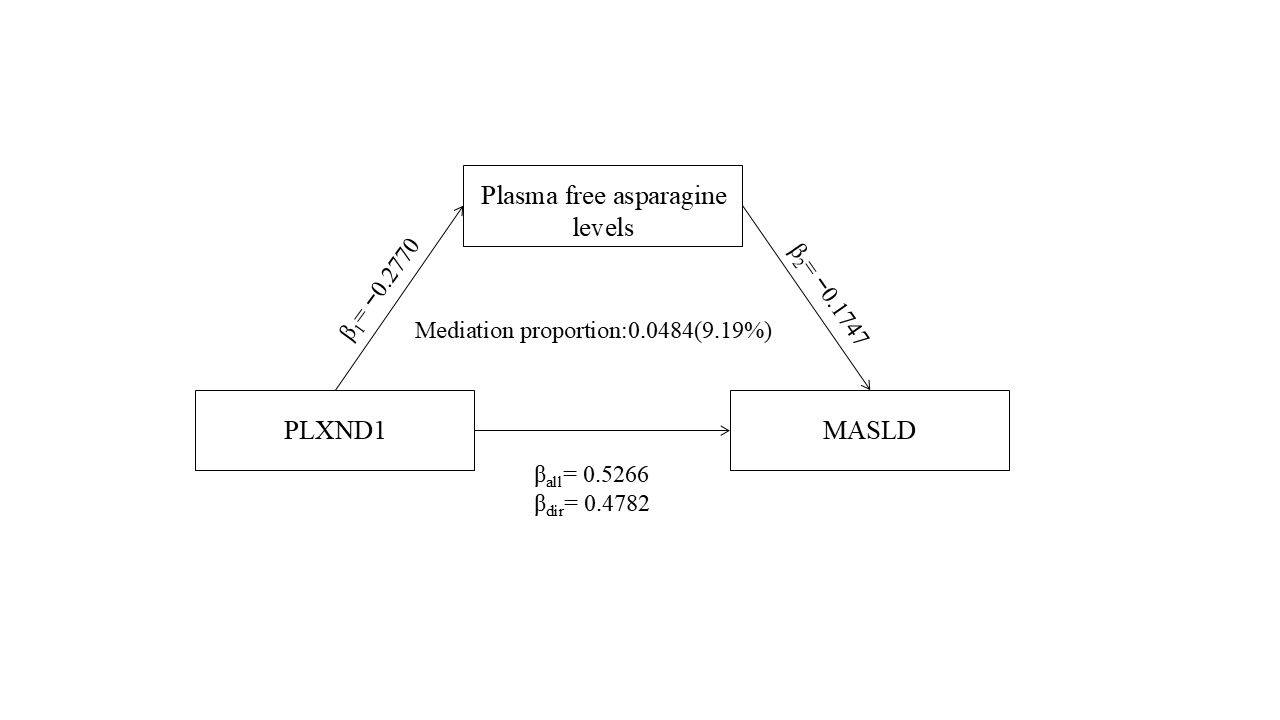

Supplement: Supplementary file 1 [file metabolites-16-00378-s001.zip › File S1. PLXND1-中介效应分析/幻灯片5.TIF]

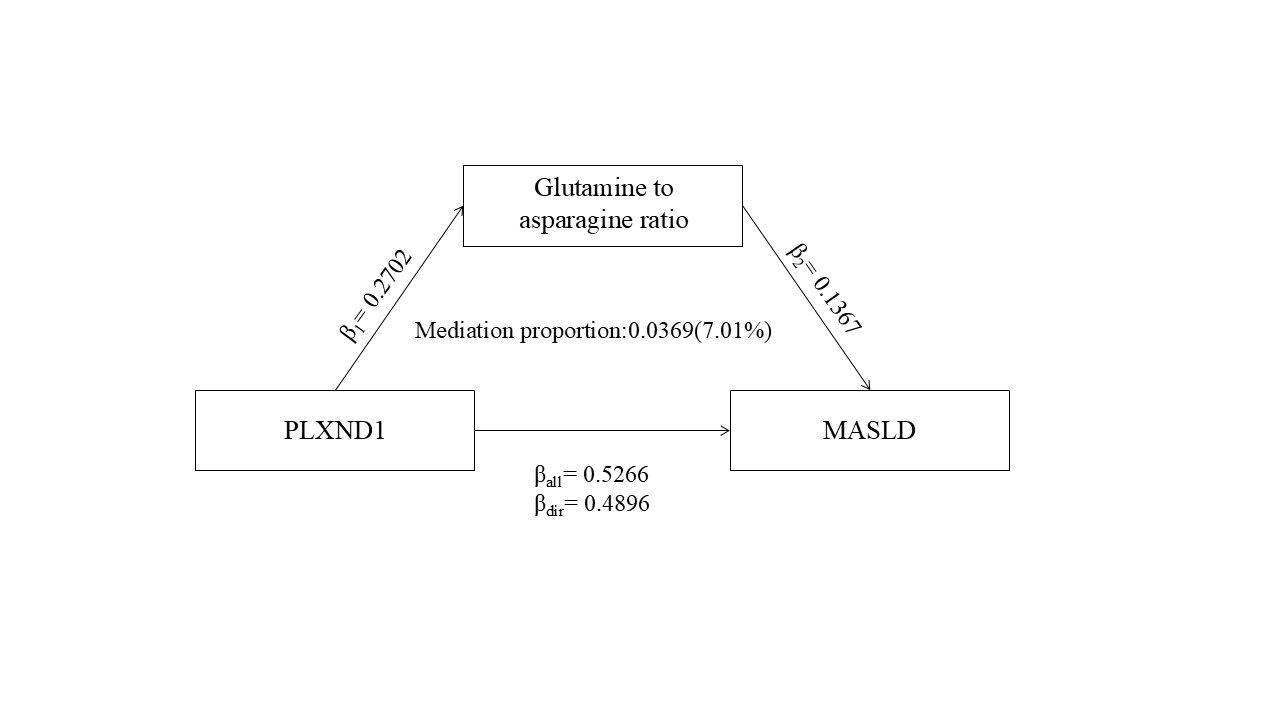

Supplement: Supplementary file 1 [file metabolites-16-00378-s001.zip › File S1. PLXND1-中介效应分析/幻灯片6.TIF]

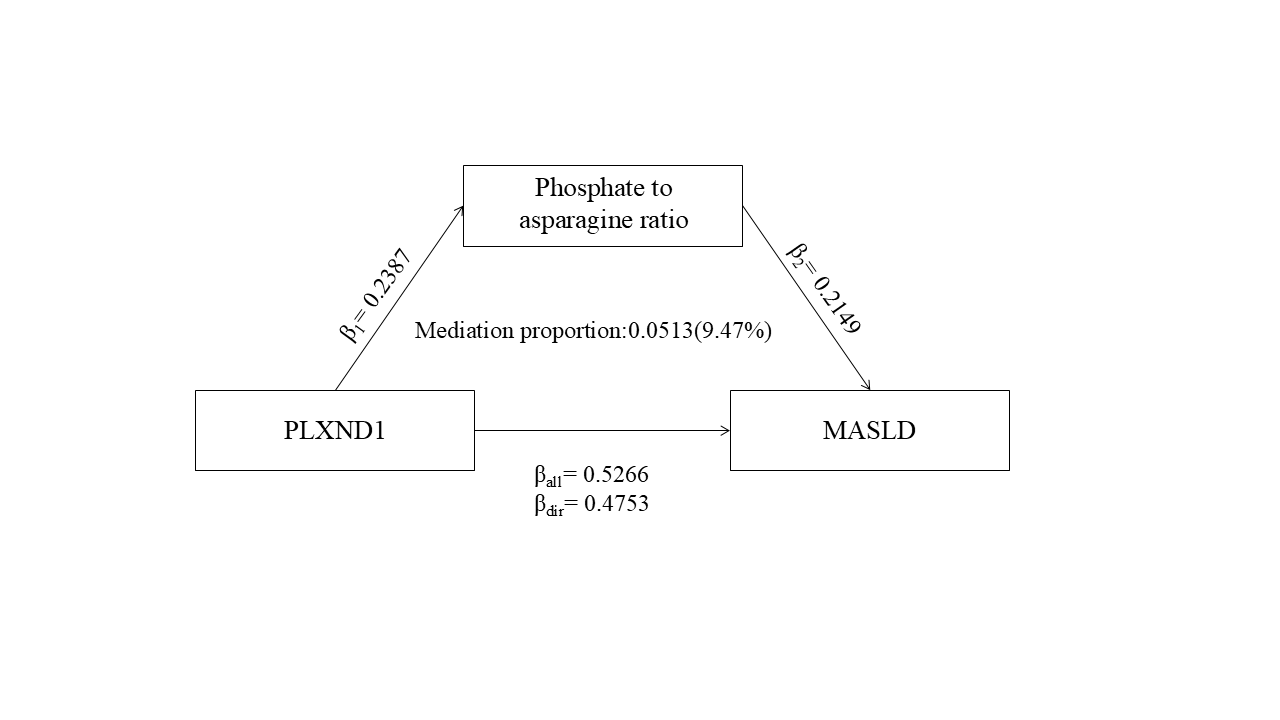

Supplement: Supplementary file 1 [file metabolites-16-00378-s001.zip › File S1. PLXND1-中介效应分析/幻灯片7.TIF]

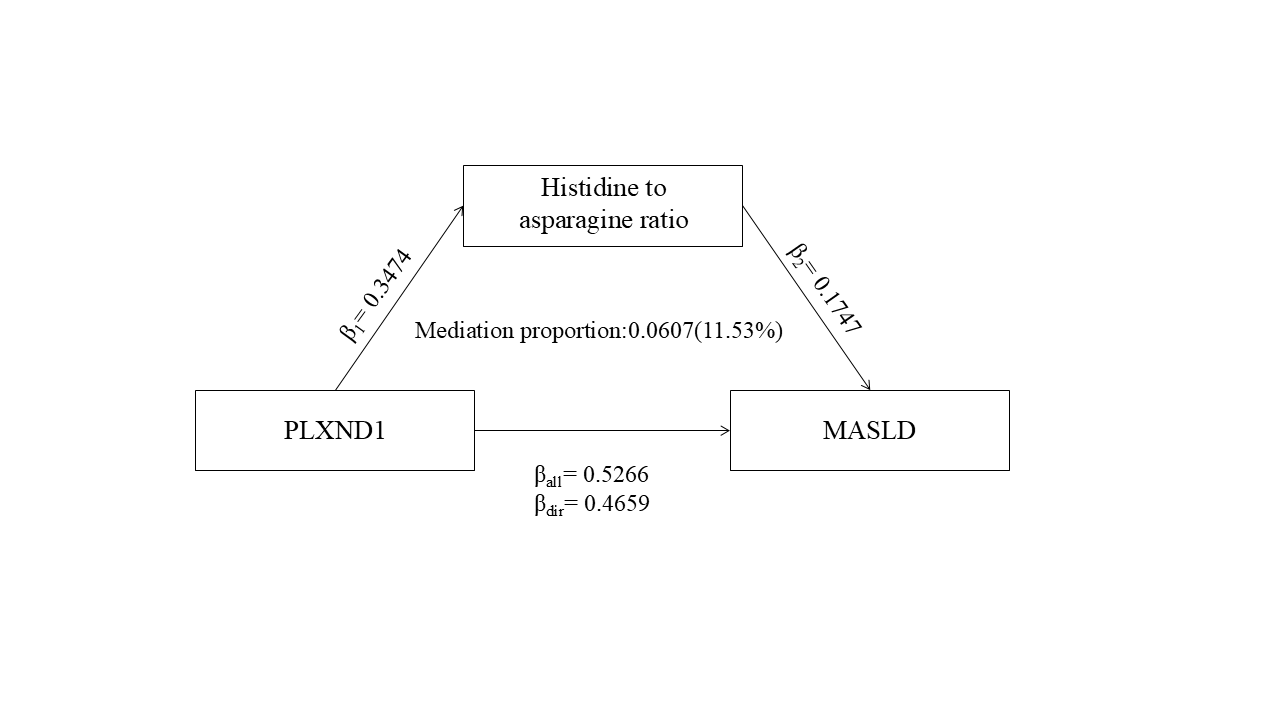

Supplement: Supplementary file 1 [file metabolites-16-00378-s001.zip › File S1. PLXND1-中介效应分析/幻灯片8.TIF]
